# The six blind men and the elephant: Are episodic memory tasks tests of different things or different tests of the same thing?

**DOI:** 10.1016/j.jecp.2015.03.006

**Published:** 2015-09

**Authors:** Lucy G. Cheke, Nicola S. Clayton

**Affiliations:** Department of Psychology, University of Cambridge, Cambridge CB2 3EB, UK

**Keywords:** Episodic memory, What–Where–When test, Free recall, Cued recall, Source memory, Unexpected question

## Abstract

The development of episodic memory in children has been of interest to researchers for more than a century. Current behavioral tests that have been developed to assess episodic memory differ substantially in their surface features. Therefore, it is possible that these tests are assessing different memory processes. In this study, 106 children aged 3 to 6 years were tested on four putative tests of episodic memory. Covariation in performance was investigated in order to address two conflicting hypotheses: (a) that the high level of difference between the tests will result in little covariation in performance despite their being designed to assess the same ability and (b) that the conceptual similarity of these tasks will lead to high levels of covariation despite surface differences. The results indicated a gradual improvement with age on all tests. Performances on many of the tests were related, but not after controlling for age. A principal component analysis found that a single principal component was able to satisfactorily fit the observed data. This principal component produced a marginally stronger correlation with age than any test alone. As such, it might be concluded that different tests of episodic memory are too different to be used in parallel. Nevertheless, if used together, these tests may offer a robust assessment of episodic memory as a complex multifaceted process.

## Highlights

• Many different episodic memory tests were compared in the same sample of 3–6 year-old children.
• Age-independent correlation was low, suggesting that these tests should not be treated as equivalent.
• A single principal component best explained variance in the dataset.
• These test should not be used in parallel, but should be adapted as a battery.

## Introduction

Six blind men wanted to discover for themselves the nature of an elephant. Each one went to the elephant and touched it. The first touched the elephant’s leg and said “it is like a tree,” the second touched the elephant’s tail and said “it is like a rope,” the third touched the elephant’s trunk and said “it is like a snake,” the fourth touched the elephant’s tusk and said “it is like a spear,” the fifth touched the elephant’s side and said “it is like a wall,” and the sixth touched the elephant’s ear and said “it is like a fan.”

Ancient Indian fable

Characterizing healthy episodic memory development in young children is important because it allows problems with memory to be identified and informs appropriate educational strategies. Although the development of memory in children has been studied for nearly a century, to date there is considerable variation in the methodologies used to do so. The fable of the six blind men and the elephant serves to warn us that a single perspective on an intangible phenomenon may provide truth but can also be misleading. As psychologists, we can never directly assess psychological processes but can only measure performance on particular tests that are thought to rely on those processes. Different tests of episodic memory stem from different philosophical, theoretical, and empirical origins, and they differ substantially in the outward behavior they assess. Such eclecticism can be both a strength and a weakness. A range of testing methodologies can allow triangulation on a single common feature. This may allow production of a battery of measures that provides a more complete picture of a psychological process. However, a range of tests that vary largely in their methodologies may merely muddy any possible interpretation.

In this study, the same sample of 3- to 6-year-old children was tested on a range of episodic memory tests. These tests are all very different in their surface features, so it might be predicted that they would produce different results. Nevertheless, they all putatively assess the same underlying cognitive ability, and as such it might instead be predicted that there should be a demonstrable association among them, reflecting this latent variable. The tests we chose to investigate are some of those that have been claimed to tap episodic memory or are candidates for such a claim. Therefore, we should expect to see a similar developmental change in all of the tests (Wellman, Cross, & Watson, 2001). In the following section, we briefly review the literature concerning these tasks.

### Free and cued recall

Free and cued recall paradigms involve learning a series of items (words or pictures) and then later being asked to recall them, either with (cued) or without (free) external cues such as category words to aid recollection. Freely recalled items are more likely to be reported as “remembered” rather than as “known” compared with cued items (Tulving, 1985) and, therefore, are considered to be more reliant on episodic memory. Both free recall and cued recall improve between 3 and 8 years of age, with children of all ages reliably finding cued recall to be the easier of the two (Naito, 2003; Perner & Ruffman, 1995; Sluzenski, Newcombe, & Ottinger, 2004).

### What–Where–When

The What–Where–When test requires participants to remember the time and location of a particular event. Clayton and Dickinson (1998) argued that this requires an integrated spatiotemporal representation of the event, which corresponds to Tulving and colleagues’ definition of episodic memory (Tulving, 1972). The What–Where–When test produces cross-sectional developmental patterns similar to those of other tests, with improvements between 2.5 and 5 years of age (Burns, Russell, & Russell, in press; Hayne & Imuta, 2011; Newcombe, Balcomb, Ferrara, Hansen, & Koski, 2014; Russell, Cheke, Clayton, & Meltzoff, 2011).

### Unexpected source memory

Source memory tests assess retention of the context of an event rather than its focal targets (Wheeler, Stuss, & Tulving, 1997). Here, participants are required to report not only what was learned but also (and unexpectedly) on the details of the context in which the learning occurred. Zentall and colleagues argued that deliberate encoding reduces the contribution of episodic memory, and thus only a question that is unexpected requires an individual to episodically reexperience the original event (Zentall, Clement, Bhatt, & Allen, 2001; Zentall, Singer, & Stagner, 2008). However, Ornstein, Haden, and Elischberger (2006) argued that it is children’s growing competence with event recall that translates into increasingly deliberate encoding. This would suggest that episodic memory development may facilitate the later emergence of purposeful remembering (Cuvo, 1975). Thus, it is unclear whether younger children may encode “expected” and “unexpected” items differently. Children under 5 years have, however, demonstrated very poor source memory (Drummey & Newcombe, 2002; Gopnik & Graff, 1988; Whitcombe & Robinson, 2000).

Absent from our analysis in this article is a consideration of autobiographical memory reports. However, to the extent that they require children to report on previous events, unexpected/source memory tests can be viewed as similar in some ways to lab-based autobiographical/event memory tests. Autobiographical memory has been studied extensively in young children (e.g., Fivush, 2014; Goodman, Ogle, McWilliams, Narr, & Paz‐Alonso, 2014; Reese, 2014). Much of this work has examined children’s verbal recall of events during parent–child conversations (e.g., Burch, Austin, & Bauer, 2004; Farrant & Reese, 2000; Haden, Ornstein, Rudek, & Cameron, 2009) and interviews elicited by experimenters (e.g., Ornstein, Gordon, & Larus, 1992). Fivush (2011) and Fivush and Nelson (2004) argued for a distinction between episodic memory and autobiographical memory, suggesting that the former should be characterized by the content of the memory, whereas the latter requires an additional layer of cognitive sophistication. The current study took as its starting point this more “minimalist” view of episodic memory (see also Clayton & Russell, 2009) and as such does not involve an assessment of autobiographical memory.

In summary, the literature using different episodic memory tests suggests that performance on each improves between 3 and 6 years of age. This may mean that these tests are able to produce consistent developmental trajectories despite very different testing methodologies. However, establishing comparable improvement in test performance throughout the preschool years is not sufficient evidence to conclude that a common cognitive process underlies performance on these different tasks. After all, many cognitive (e.g., theory of mind, language) and non-cognitive (e.g., running speed, height) factors improve over this period. What is required is an assessment of the degree to which performances on these different tests are related within the same individuals. This type of investigation has been carried out with respect to different tests of prospection, finding good correlation among most tests (Atance & Jackson, 2009).

Here, 3- to 6-year-old children were presented with three putative tests of episodic memory (What–Where–When, Unexpected Source Memory, and Free Recall) and one that is thought to rely less on episodic memory than on semantic memory (Cued Recall) (Tulving, 1985). All of the memory tests were designed to produce continuous data (not pass/fail). This enabled us to investigate whether performances on these tests are correlated and whether they are able to hold together as a “battery” of tests.

## Method

### Participants

At total of 106 children between 36 and 83 months of age were recruited from schools and nurseries in the Cambridge area of England. There were 27 3-year-olds (*M* = 42.3 months, *SD* = 3.6), 18 4-year-olds (*M* = 53.67 months, *SD* = 3.5), 27 5-year-olds (*M* = 66.2 months, *SD* = 3.3), and 34 6-year-olds (*M* = 76.7 months, *SD* = 2.7). The sample consisted of 49 girls and 57 boys (3-year-olds: 15 girls and 12 boys; 4-year-olds: 6 girls and 12 boys; 5-year-olds: 15 girls and 12 boys; 6-year-olds: 13 boys and 21 girls). The study was approved by the Cambridge University psychological research ethics committee. Informed written consent was received from parents before any child took part. Testing took place in an empty room or in a quiet corner of a classroom in the school/nursery. The majority of the children were native English speaking, Caucasian, and middle class, representative of the local area.

### Procedure

**Fig. 1:**
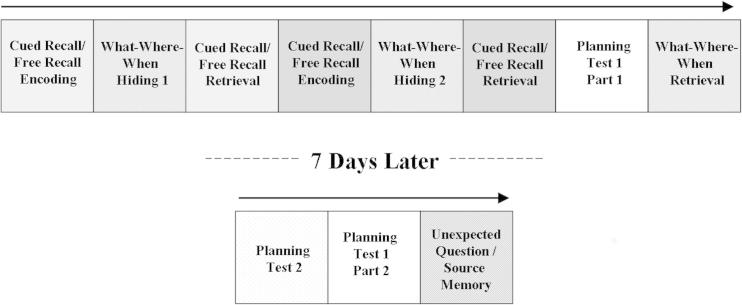
Schematic of experiment order. “Planning test” is an experiment reported elsewhere.

As shown in Fig. 1, the study had a nested design in which elements of each test described here formed the retention intervals for the other tests.

#### Free Recall and Cued Recall

In both recall tasks, children were shown eight photos of familiar objects or animals (e.g., a book, a horse) and were asked to name each one in turn. They were then told to look at the images and try to remember what was in them. Recall occurred after a delay of approximately 5 min. In Free Recall children were asked to tell the experimenter “what pictures had been on the cards,” whereas in Cued Recall they were asked to tell the experimenter what pictures of specific categories (animals or toys) had been on the cards. This methodology followed that of Perner and Ruffman (1995), but the images were not the same.

#### What–Where–When

**Fig. 2:**
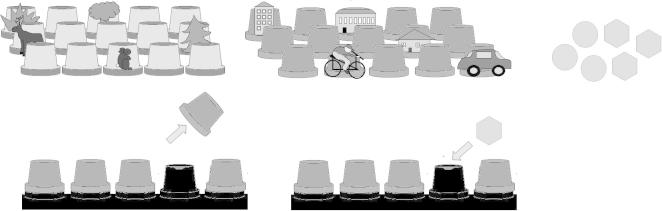
Illustration of the What–Where–When test. The two locations were the “forest” (left) and the “town” (right), and the items were “gold” and “silver” treasure. Items could be hidden under the pots, as illustrated at the bottom of the figure.

Fewer children took part in this task because children were split between this and another study (not reported). As such, 68 children took part in this task (32 girls and 36 boys; 12 3-year-olds, 13 4-year-olds, 17 5-year-olds, and 26 6-year-olds). As shown in Fig. 2, children were given three pieces of “gold treasure” (plastic £1 coins) and three pieces of “silver treasure” (plastic 20p coins) and asked to hide them in two different trays (the “forest” and the “town”). There were two hiding sessions separated by approximately 5 min. In each session, children could hide in only one tray. During hiding, the experimenter highlighted each coin’s identity by saying, “Where are you going to hide that [gold/silver] treasure?” After a delay (∼5–10 min), a new character (“Mr. Crow”) who had “stolen” a specific subset of the hidden coins was introduced; for example, the gold treasure from the second hiding session had been stolen. Children were explicitly informed which treasure remained (e.g., “the GOLD treasure from BEFORE we looked at cards”). All elements of the treasure that was left (particularly the “when” element) were described to children in a number of ways (e.g., “before,” “earlier,” “first,” “longer ago”) to increase their chances of understanding what was being asked. Children were then asked to indicate the location of the remaining treasure by pointing. Children could then swap the coins for a sticker.

#### Unexpected Source Memory

One week after the first stage of the experiment, children were unexpectedly asked about elements of the “games” that had been played. The 11 questions concerned contextual details about the learning episode. Both open-ended questions (e.g., “What animal stole the treasure in the pirate game?”) and cued-choice questions (e.g., “Which video had a teddy bear in it?”) were asked. Some referred to games children had played that day, and others referred to games they had played the previous week.

## Results

Data were analyzed using Pearson’s and partial correlations to measure covariation among various test performance. To assess whether performance on the different tests may reflect a single latent variable, principal component analysis was used. Alpha was set at .05.

Preliminary analysis revealed no differences in performance between boys and girls, and therefore gender was not considered in the main analyses. Table 1 shows the mean, standard deviation, and range of scores for each age group on each of the tests.

**Fig. 3:**
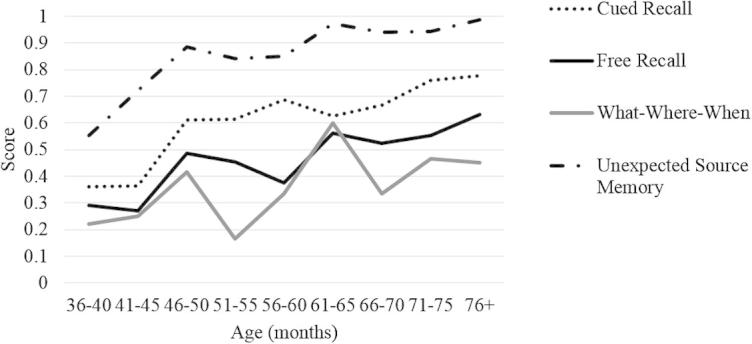
Improvement on each task with age.

As shown in Fig. 3, performance on all tests was positively associated with age. Table 2 indicates the correlations with age as well as the associations among the tests.

Performances on many of the memory tests were related. The significant correlation between Unexpected Source Memory and Free Recall remained when Cued Recall was controlled (*r* = .317, *p* = .02). The positive correlation between Free Recall and Cued Recall remained after age was controlled. However, all of the other correlations were reduced to non-significance when controlling for age.

The central question of this study concerns whether different tests of episodic memory can be said to be assessing the same underlying cognitive process. One way of addressing this is to examine the extent to which an appropriately amalgamated score from all four tests (using the 68 children who took part in all four tests) is able account for more variance than any of the tests on their own. This was done in two stages, a covariance summary and then a prediction of a known relevant variable, as a validation paradigm. A single principal component was produced by this analysis and had an eigenvalue of 1.91, explaining 47.9% of total variance. No other component had an eigenvalue over 1.0, and orthogonal rotation did not increase the eigenvalue of the second principal component over 1.10, indicating a single underlying source of covariance in this dataset. Of the four tests, the What–Where–When test loaded least well into this factor (.340). Finally, the principal component was then correlated with age. This produced a marginally stronger correlation than any of the individual tests (*r* = .699). This correlation was similar when dropping the weakest loading test (What–Where–When) and re-extracting the first principal component (*r* = .708).

## Discussion

The aim of this experiment was to investigate the consistency of a number of different tests putatively assessing the same underlying psychological process, namely episodic memory. Performance on each of the four tests was shown to improve gradually between 3 and 6 years of age, in line with previous literature (e.g., Hayne & Imuta, 2011; Naito, 2003; Reese, 2014). These tests differ in their surface features but are conceptually similar in that all aim to assess episodic memory. As would be predicted by this similarity, performances on many of the tests were correlated; however, few correlations remained significant after covariation due to age-related improvement being controlled. This result demonstrates that these tests are *not* equivalent and should not be treated as so when assessing episodic memory in children. However, the principal component analysis suggests that a single underlying factor may satisfactorily fit the data. Thus, there may be mileage in adapting these tests with the aim of reducing their surface differences and bringing them together into a single battery.

Nevertheless, there is still some distance to go before a satisfactory battery of episodic memory tasks can be achieved. The tests used in this study were not designed to be methodologically similar; rather, they were designed to represent the variation present between currently used tests. Therefore, it is difficult to identify what factors contribute to low levels of age-independent correlation. There are many non-mnemonic cognitive and non-cognitive abilities that develop across the age range covered here. Furthermore, the tests differed in the extent to which they required receptive and productive verbal competence, confidence around a strange experimenter, executive functions, and other “extra-target” challenges. However, recent work with adults (Cheke & Clayton, 2013) shows that poor correlations among these tests are present even during adulthood. This implies that development of extra-target factors might not provide a full explanation for low covariation.

Each of the tests used in this study explicitly tests different mnemonic skills. The What–Where–When test requires binding of spatiotemporal features. The Unexpected Source Memory task requires the ability to reanalyze previous experiences for new information. The Free Recall task assesses the ability to mentally initiate and guide retrieval in the absence of external cueing, and the Cued Recall task requires the ability to use category words as retrieval cues. These different elements are by no means the only important features of episodic memory: One limitation of this study is the absence of a measure of these children’s autobiographical memory reports. Still, the tests employed cover a range of different perspectives on the “defining features” of episodic memory. Adaptation of these types of test to facilitate the creation of a battery could provide benefits that are greater than the sum of it’s parts. Ultimately, it may allow the assessment of episodic memory as a *whole* without undue emphasis on any one particular feature.

To summarize, this study revealed few associations for performance on different tasks putatively assessing episodic memory among 3- to 6-year-old children when age was controlled. This suggests that these tests are sufficiently different to lead to disparate results in studies across the episodic memory literature. Nevertheless, it was also found that performance across the tasks was well described by a single factor model. This may indicate that although the different tests are too different to use independently as equivalent tests, the conceptual similarities are sufficient to warrant their adaptation to create an episodic memory battery. Like the blind men’s perspective of the elephant, this would have informative powers above and beyond each individual test. Future work, therefore, should focus on development of such a battery in which the tests are more methodologically matched but remain structurally distinct. This battery might further include autobiographical reports and tests of episodic foresight. In this way, researchers will be able to investigate episodic memory not only as a coherent single process but also as a multifaceted one.

## Figures and Tables

**Table 1:** Mean performance, standard deviation and range of scores for each task for each age group.

Age (Years)	Free Recall		Cued Recall		Unexpected Source		What–Where–When	
	Mean (*SD*)	Range	Mean (*SD*)	Range	Mean (*SD*)	Range	Mean (*SD*)	Range
3-Years	.30 (.20)	0–.50	.33 (.26)	0–.75	.54 (.32)	.10–.80	.28 (.28)	0–.67
4-Years	.47 (.23)	.125–1	.65 (.15)	.50–1	.90 (.06)	.80–1	.23 (.21)	0–.67
5-Years	.52 (.17)	.125–.75	.68 (.17)	.375–1	.93 (.09)	.80–1	.41 (.32)	0–1
6-Years	.60 (.10)	.375–.75	.78 (.14)	.50–1	.95 (.08)	.70–1	.47 (.30)	0–1

**Table 2:** Correlations among memory tests and between memory tests and age, with partial correlations controlling for age in parentheses.

	Free Recall	Cued Recall	Unexpected Source	What–Where–When
Age	.539⁎⁎⁎	.632⁎⁎⁎	.617⁎⁎⁎	.268⁎
Free Recall	–	.544⁎⁎⁎ (.312⁎⁎⁎)	.396⁎⁎⁎ (.105)	.172 (.048)
Cued Recall	–	–	.422⁎⁎⁎ (.066)	.323⁎⁎ (.205)
Unexpected Source	–	–	–	.085 (.001)

*Note.* Numbers represent Pearson’s *r* values.

⁎ *p* < .05

⁎⁎ *p* < .01

⁎⁎⁎ *p* < .001
